# *Juniperus oxycedrus* L. ssp. Essential Oil Microneedles: A Promising Antimicrobial and Wound Healing Activity

**DOI:** 10.3390/ph17010040

**Published:** 2023-12-27

**Authors:** Basem Battah, Lama Shbibe, Osama Ahmad, Chadi Soukkarieh, Souad Mahmoud Al Okla, Teresa Chianese, Luigi Rosati, Lalitkumar K. Vora, Li Zhao, Alessandra Marrazzo, Marco Ferrari, Linlin Li, Ryan F. Donnelly, Stefania Zanetti, Vittorio Mazzarello, Matthew Gavino Donadu

**Affiliations:** 1 Department of Biochemistry and Microbiology, Faculty of Pharmacy, Antioch Syrian Private University, Maaret Saidnaya 22734, Syria; 2 Faculty of Science, Damascus University, Damascus P.O. Box 30621, Syria; menaayoub1990@gmail.com (L.S.); soukkarieh@gmail.com (C.S.); 3 Faculty of Medicine, Syrian Private University (SPU), Daraa International Highway, Damascus 36822, Syria; osamaahmad62@gmail.com; 4 Faculty of Medicine, Kalamoon University, Damascus 222, Syria; 5 College of Medicine and Health Sciences, National University of Science, Sohar 321, Oman; souadalokla@nu.edu.om; 6 Department of Biology, University Federico II, Via Cintia 21, 80126 Napoli, Italy; teresa.chianese2@unina.it (T.C.); luigi.rosati@unina.it (L.R.); 7 Medical Biology Centre, School of Pharmacy, Queen’s University Belfast, 97 Lisburn Road, Belfast BT9 7BL, UK; l.vora@qub.ac.uk (L.K.V.); llizhaoz@hotmail.com (L.Z.); linlinli@gmail.com (L.L.); r.donnelly@qub.ac.uk (R.F.D.); 8 Hospital Pharmacy, Azienda Ospedaliero Universitaria di Sassari, 07100 Sassari, Italy; a.marrazzo@studenti.uniss.it; 9 Institute for Maternal and Child Health—IRCCS Burlo Garofolo, Via dell’Istria, 65, 34137 Trieste, Italy; dr.marcoferrari@gmail.com; 10 Department of Biomedical Sciences, University of Sassari, 07100 Sassari, Italy; stefania.zanett_2020@libero.it (S.Z.); vmazza@uniss.it (V.M.); 11 Scuola di Specializzazione in Farmacia Ospedaliera, Department of Medicine, Surgery and Pharmacy, University of Sassari, 07100 Sassari, Italy; mdonadu@uniss.it; 12 Hospital Pharmacy, Giovanni Paolo II Hospital, ASL Gallura, 07026 Olbia, Italy

**Keywords:** skin infection, essential oil, juniper, antimicrobial, wound healing

## Abstract

The use of essential oil (EO) in treating infected wounds is still challenging. A lot of effort has been made to make such an application more convenient. Recently, microneedles (MNDs) have been considered as a smart dermal delivery system to overcome the poor absorption and distribution, low bioavailability, and skin penetration of some drugs. The aim of our study is to evaluate the wound healing activity of juniper-EO-loaded MNDs (EO MNDs) against wounds with bacterial and fungal infection. The Polyvinylpyrrolidone (PVP) MNDs were prepared using the gel-filled mold technique and loaded with juniper EO. In vivo models were created and wounds on rats were infected with two clinically isolated bacterial strains *Pseudomonas aeruginosa* and *Staphylococcus aureus*. Furthermore, *Candida albicans* was used to mimic fungal infection and juniper EO MNDs were tested. The obtained results showed an improvement in wound healing which started from the third day after application of the juniper EO MNDs, and at the sixth day post-infection, the treated wounds were significantly smaller than untreated wounds. A complete healing was shown by the 12th day after infection. Furthermore, our cytotoxicity results showed a cytotoxic effect of juniper EO MNDs on epithelial cells, which explained the faster wound healing in rats. Our study showed that juniper EO MNDs represent a novel strategy in EO delivery with minimal invasion. Juniper EO MNDs demonstrated significant antimicrobial activity against both the bacterial strains *Pseudomonas aeruginosa* and *Staphylococcus aureus* and against one fungal strain, *Candida albicans*. Finally, application of juniper EO MNDs exerted promising activity in the treatment and healing of wound infection.

## 1. Introduction

The healing of skin injuries usually occurs quickly through an orderly process involving inflammation, proliferation, and remodeling phases. However, wounds can become infected, hindering the healing process and making it challenging to treat infected wounds [1]. Diabetes and other conditions that impair wound healing can elevate the risk in patients, fostering the formation of chronic infections. Conditions such as diabetes mellitus and skin infections significantly impede the wound healing process, making the treatment of infected wounds both challenging and less effective. This difficulty in achieving effective healing poses a direct threat to the patient’s life [2].

Bacterial infection is a main factor that contributes to chronic wound pathogenesis and complicating the healing process. Despite all effort, the treatment and healing of chronically infected wounds remain challenging [3]. Substantial research has been conducted to study the factors contributing to chronic infections and to develop improved treatment strategies. For example, wound exudates can reduce the local bioavailability of the topically applied therapeutics, diminishing therapeutic efficacy [4]. Addressing infected wound treatment remains a significant clinical challenge. It demands not only the eradication of pathogens but also the repair and safeguarding of the wound from subsequent infections. To meet these needs, microneedles (MNDs) have been developed as a sophisticated, smart delivery system tailored for targeted drug administration to wounds [4,5]. The rise of nanomedicine, along with the incorporation of nanotechnology and nanomaterials, has marked a significant advancement in this area, witnessing a recent upsurge in application. Essential oils (EOs), products of natural origin, have shown promising potential for clinical use [6]. The antimicrobial properties of EOs, both antibacterial and antifungal, have been extensively researched and are well-established [7,8]. It has been proposed that EOs interfere with bacterial proliferation through multiple mechanisms: (1) compromising the bacterial cell membrane’s phospholipid bilayer; (2) increasing cell membrane permeability, which leads to the leakage of potassium ions and protons; (3) allowing the escape of critical intracellular substances such as nucleic acids and proteins; (4) altering fatty acid composition; and (5) inducing cell lysis [9].

Recently, MNDs have proven successful in the topical delivery of drugs, increasing drug bioavailability in the wound bed [10]. MNDs are considered as an advanced physical tool for transdermal and intradermal applications, enhancing the delivery of active ingredient and cells topically [11]. MNDs can be fabricated using various materials, including polymers [12], silicon [13], metal [14], and glass [15]. Therefore, MNDs are commonly categorized based on their materials and serve distinct purposes. Solid MNDs, produced from silicon and metals, offer mechanical properties without drug incorporation. In contrast, dissolving MNDs integrate a biodegradable matrix with the drug to ensure minimal waste post-application. However, polymeric MNDs present several disadvantages compared to their inorganic counterparts, including challenges related to biocompatibility, biodegradation, and non-toxicity.

Furthermore, challenges remain regarding material selection and stability [16]. Biocompatibility is essential for the effective delivery of active materials. Biodegradable materials such as water-soluble and biodegradable polymers are considered as the optimal choice for facilitating appropriate delivery [17]. Biodegradable MNDs offer the advantage of delivering active materials while safely minimizing immunogenic reactions and accommodating a large quantity of the active ingredient or drugs [18]. Reducing bacterial load at the site of the infected wound through direct antimicrobial application on infected wounds is a fundamental approach needed to provide successful wound healing and rapid closure [19]. The antimicrobial and antifungal properties of EOs are well documented. Juniper EO has been extensively studied.

The antibacterial activity of *Juniper communis* EO was demonstrated against *Staphylococcus aureus* (*S. aureus*), *Streptococcus pyogenes* (*S. pyogenes*) from Gram-positive bacteria, and *Escherichia coli* (*E. coli*), and *Pseudomonas aeruginosa* (*P. aeruginosa*) from Gram-negative bacteria, with more activity against Gram-positive than Gram-negative bacteria [20]. In addition, juniper EO exerted a relevant antifungal activity against Candida albicans (*C. albicans*) [21]. Furthermore, essential oil from juniper berry (*Juniper communis* L., cupressacae) has demonstrated significant anti-Gram-negative, anti-Gram-positive, and antifungal activities. The minimum inhibitory concentration (MIC) values ranged between 8 and 70% (*v*/*v*) against Gram-positive bacteria like *S. aureus* (15% *v*/*v*) and Gram-negative bacteria like *Salmonella enteritidis* (*S. enteritidis*) (70% *v*/*v*), and were below 10% against fungal strains, with (1% *v*/*v*) against *C. albicans* [22].

The delivery of antibacterial EOs has various advantages, including direct application to the infected wound site, rapid onset of action, avoidance of first-pass metabolism through gastrointestinal absorption, being safe and painless, and high bioavailability at the target site [23]. With their ability to efficiently deliver therapeutic payloads, MNDs represent a promising platform for juniper EO wound treatment. In this study, capitalizing on these benefits and recognizing the potential antibacterial activity of juniper EO, we developed EO-loaded dissolving polymeric MNDs and evaluated their wound healing and antibacterial efficacy. The wound healing and the direct antimicrobial activity of the juniper EO MNDs were tested against wounds infected with Gram-positive and Gram-negative bacteria well known to cause serious acute and chronic skin infections. Furthermore, a fungal wound infection model was employed to evaluate the efficacy of juniper EO MNDs against fungal skin infections.

## 2. Results

### 2.1. Chemical Characterization of Essential Oil Juniperus L. ssp. Macrocarpa

We calculated the response factors (RFs) for the primary analyte using 2,6-dimethylphenol as a reference and extended these factors to other analytes based on similarities in functional groups or structure. To mitigate the effects of trace contaminants, we crafted a response factor solution containing four or five substances, including 2,6-dimethylphenol. Sensitivity towards the flame ionization detector was lower for oxygenated compounds compared to hydrocarbons. The RFs were derived from a standard mixture comprising α-terpineol, α-pinene, neral, geranyl acetate, geranial, and caryophyllene. In this blend, terpenes represented 92%, aldehydes approximately 5%, and alcohols, esters, and sesquiterpenes accounted for about 1% each. Our findings revealed RF values of 1 for hydrocarbons, 0.71 for esters, and 0.80 for alcohols, with correction factors of 1.24 for aldehydes and ketones, 1.408 for esters, and 1.28 for alcohols. The predominant organic compounds identified were ß-pinene (13.42% ± 0.09), α-pinene (56.63% ± 0.24), and limonene (56.63% ± 0.24), as listed in the Supplementary Materials (Table S1).

### 2.2. Temporal Killing Activity of Juniper MNDs against P. aeruginosa, S. aureus and C. albicans

**Figure 1:**
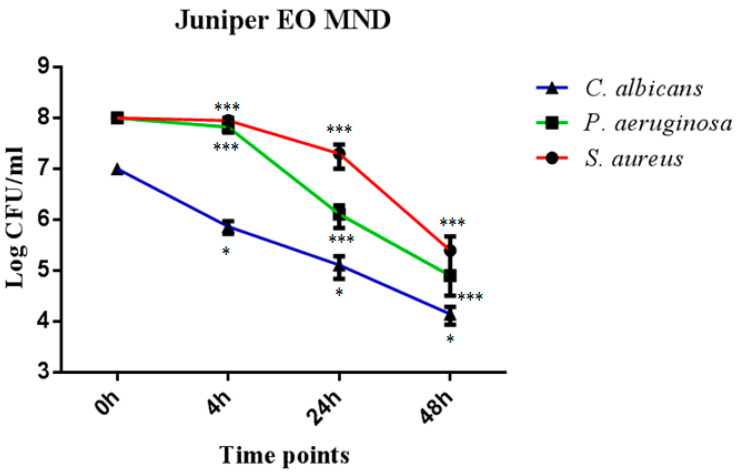
Temporal killing activity of juniper EO MNDs against three different microbial strains. Colony forming units (CFUs) were evaluated following the incubation of *S. aureus*, *P. aeruginosa*, and *C. albicans* in the presence of juniper EO MNDs, with evaluations conducted at 4 h, 24 h, and 48 h time points. The figure shows that juniper EO MNDs exhibited a significant reduction in CFUs of *C. albicans* at three time points (* *p* < 0.05). Similarly, a remarkable reduction in CFUs was observed for *P. aeruginosa* at 4 h, 24 h, and 48 h (*** *p* < 0.001). In the case of *S. aureus*, a substantial decrease in CFUs occurred after 24 h and 48 h of incubation (*** *p* < 0.001). The data presented represent a single experiment replicated three times.

**Figure 2:**
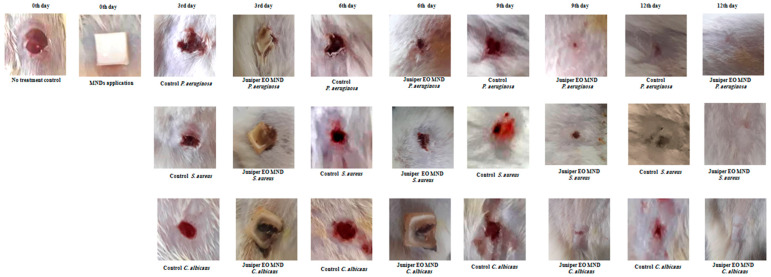
Healing progression in infected rat wounds before and after juniper EO MNDs treatment. Illustrative images depict the healing trajectory of rat wounds infected with *P. aeruginosa*, *S. aureus*, and *C. albicans* before and after juniper EO MNDs treatment. Following infection, MNDs loaded with juniper EO were applied, and healing was monitored at 3, 6, 9, and 12 days, compared to negative control (no treatment) and positive control (treated with gentamicin and fluconazole) groups (Figure 3). The images show consistent improvement in wound healing at all time points. By day 12, MND-treated wounds were fully healed, contrasting with unrepaired wounds in the negative control and partially healed wounds in the positive control groups. Data represent three experiments for three microorganisms, each replicated three times.

In order to evaluate the direct killing activity of juniper EO MNDs against *P. aeruginosa*, *S. aureus*, and *C. albicans*, colony forming units (CFUs) were evaluated at different time points of incubation (4 h, 24 h, and 48 h). The results showed a significant reduction in CFUs for all three microorganisms across the three time points. Specifically, juniper EO MNDs exhibited a noteworthy decrease in CFUs of *C. albicans*, ranging between 2 and 3 log CFUs at the three time points. Similarly, a substantial reduction in CFUs of *P. aeruginosa* was observed, ranging between 1 and 4 log CFUs across the evaluated time intervals. For *S. aureus*, a significant reduction in CFUs was evident at 24 h and 48 h post-infection, by 1 and 2 log CFUs, respectively, as shown in (Figure 1). These results correlate with our initial clinical observation, where the improvement started at the third day post-treatment for all three groups (Figure 2).

### 2.3. In Vivo Assessment of Wound Healing Efficacy of Juniper MNDs against Bacterial and Fungal Infections

**Figure 3:**
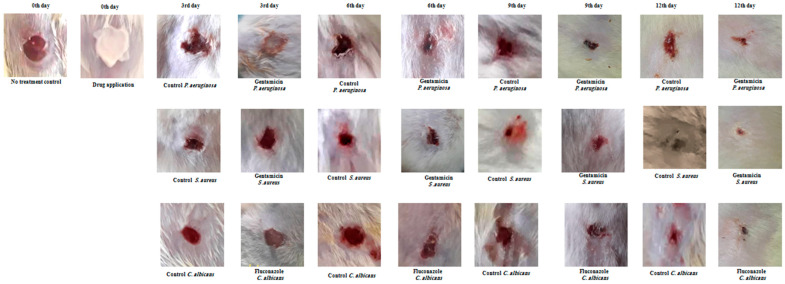
Wound healing evaluation of infected wounds in rats using topical creams. Representative images depict infected wounds in rats treated with gentamicin cream for bacterial infection and fluconazole cream for fungal infection. Wounds infected with *P. aeruginosa*, *S. aureus*, and *C. albicans* were treated, showing improved healing at various time points (3, 6, 9, 12 days) compared to the untreated control group. All wounds were healed after 12 days post-treatment but not completely closed, contrasting with unrepaired control wounds. Data represent three experiments for each microorganism, repeated three times.

Juniper EO MNDs were applied to wounds infected with diverse microorganisms mimicking bacterial (*P. aeruginosa* and *S. aureus*) and fungal (*C. albicans*) infections. The untreated infected wounds served as the negative control group (empty MNDs), while gentamicin- and fluconazole-treated wounds acted as the positive controls for bacterial and fungal infections, respectively, as shown in (Figure 3). Wound healing progress was meticulously monitored and controlled at different time points (3, 6, 9, and 12 days) post-MND application, as depicted in Figure 2 and Figure 3. The results revealed a significant enhancement in wound healing compared to the control group, evident from the third day post-treatment. By the sixth day post-infection and treatment, the wound areas were notably smaller than those in the untreated group, with accelerated repair rates. Remarkably, the patches completely biodegraded alongside the healing process. All wounds infected with the three kinds of microorganisms achieved total healing after 12 days of treatment, surpassing both the negative control groups (unrepaired) and positive control groups (incompletely closed and healed).

### 2.4. The Histological Alteration after Juniper MND Treatment of Bacterial and Fungal Infections in Wound

**Figure 4:**
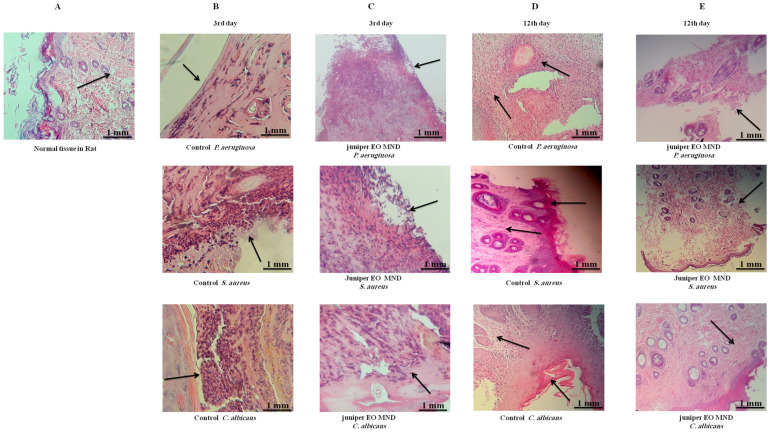
Histopathological comparison of wound healing in response to juniper EO MNDs treatment and control groups over time. The figure illustrates H&E staining of wounds infected with different microorganisms, comparing treated (juniper EO MNDs) and untreated (control) groups at different time points. (**A**) represents normal. (**B**) displays the control group (no treatment) at 3rd day after infection with *P. aeruginosa*, *S. aureus*, and *C. albicans*, showcasing acute inflammation, superficial ulceration, and pus exudates with neutrophil polymorphs infiltration. (**C**) depicts the juniper EO MNDs treatment at the same time point, demonstrating a similar inflammatory response. (**D**) shows the control group on the 12th day, revealing distinct histopathological features for each microbial infection. *P. aeruginosa* led to chronic inflammation and fibroblast proliferation. While *S. aureus* resulted in hyperkeratosis, chronic inflammation, and dense fibrosis (by scarring). The *C. albicans*-infected group showed hyperkeratosis, acanthosis, dense fibrosis, and chronic inflammation (as indicated by arrows). (**E**) At the 12th day after the juniper EO MND treatment of wounds infected with *P. aeruginosa*, *S. aureus*, and *C. albicans*, a positive response is demonstrated, with complete healing, reduced fibrosis, and scarring. Scale bar: 1 mm.

A histological examination was conducted to validate preliminary observations. The skin tissue was excised from the wound area and subjected to hematoxylin and eosin (H&E) staining. At the third day after infection with *P. aeruginosa*, *S. aureus*, and *C. albicans*, both the control (no treatment) group and the juniper MNDs group exhibited superficial ulceration, pus exudates, and neutrophil polymorph infiltration. By the 12th day post-infection, wounds infected with *P. aeruginosa* displayed chronic inflammation and fibroblast proliferation, wounds infected with *S. aureus* exhibited hyperkeratosis, chronic inflammation, and dense fibrosis, while wounds infected with *C. albicans* showed hyperkeratosis, acanthosis, dense fibrosis, and chronic inflammation. In contrast, histological studies after the 12th day of treatment revealed complete healing and the presence of normal tissue in wounds infected with *P. aeruginosa*, *S. aureus*, and *C. albicans*, as illustrated in Figure 4. These histopathological findings align with the obtained images, confirming complete healing after the 12th day of MND application. This treatment supports swift tissue recovery with fewer consequences of dysfunction, akin to first intention healing.

### 2.5. In Vitro Evaluation of Wound Healing Activity of juniper EO MNDs

Realizing the innovative importance that juniper EO MNDs could bring to the medical field, we decided to test their activity directly on epithelial cells in vitro. Therefore, two different cell lines were used: HaCaT and PNT1a. HaCaT cells are aneuploid immortal keratinocytes spontaneously transformed from adult human skin, widely used in scientific research because of their high capacity to differentiate and proliferate in vitro; in this case, this type of cells represent the first barrier with which these MNDs would come into contact. PNT1a, a human prostate lineage constituting the epithelium of the prostate gland, was chosen to further confirm the data collected with the HaCaT cells.

#### Evaluation of HaCaT Cells’ Viability after Exposure to Juniper EO MNDs

**Figure 5:**
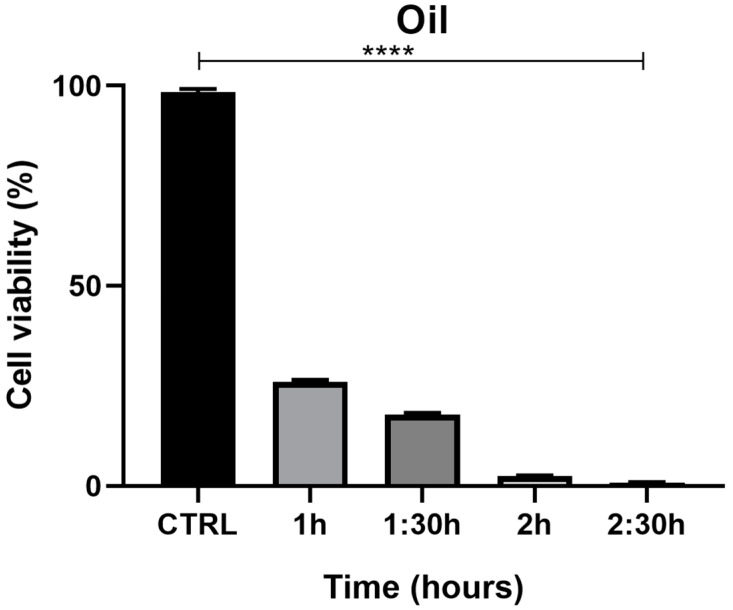
MTT assay summary for HaCaT cells exposed to juniper EO MNDs at different times: 1 h, 1.5 h, 2 h, 2.5 h. The graph illustrates cell viability reported as a percentage. Significant differences are observed among different essential oil exposure times, with higher cell viability corresponding to shorter oil exposure time and maximum cell toxicity corresponding to the longest exposure time (2.5 h). Statistical analysis was performed by analysis of variance with Bonferroni corrections (**** *p* < 0.0001).

**Figure 6:**
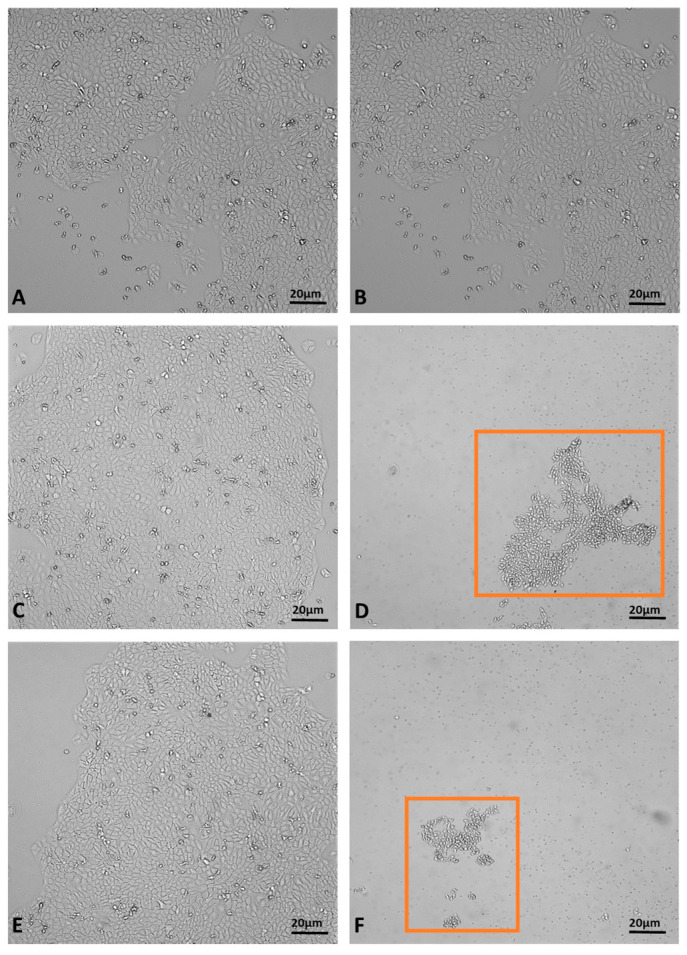
Dynamic assessment of HaCaT cells during juniper EO MNDs treatment. (**A**,**B**) Control cells at time zero and after 2.5 h. (**C**,**D**) Cells at time zero, prior to juniper EO MNDs addition, and after 1 h of treatment. (**E**,**F**) Cells at time zero, before adding juniper EO MNDs, and after 2.30 h of treatment. Orange boxes represent areas with surviving cells post-treatment. Scale bars: 20 µm.

The MTT assay, used to evaluate the effects of juniper EO MNDs on HaCaT epithelial cells, revealed a time-dependent decrease in cell viability. Indeed, at the shortest exposure time (1 h), cell viability was 27%, but decreased to 1% after 2.5 h of exposure (Figure 5. The viability percentages were calculated according to the following formula: (OD [570 nm] evaluated sample/(OD [570 nm] negative control) = R; R × 100 = % cell viability. To support the data obtained from the MTT viability test, the cells were monitored with the aid of the JuLI Stage automated cell imaging system, a time-lapse inverted microscope equipped with a camera that allows the cells to be followed over time while maintaining the ideal conditions due to the positioning of the microscope in the cell incubator, without creating further stress. Moreover, the JuLI Stage allows the same point to be photographed thanks to a function that captures a desired point and maintains it over time. The shots were taken at time zero and after the various treatment times. Images captured by the JuLI Stage inverted microscope are shown in (Figure 6). In the control, both the number of cells and their morphology did not change over time (Figure 6A,B). The presence of the biodegradable patches infiltrated with MNDs containing juniper oil impaired the cells’ condition in a time-dependent manner. Indeed, after 1 h of treatment, the number of cells decreased (Figure 6D) compared to the control (not shown); the cells that resisted the action of the juniper EO MNDs, indicated in the box, showed a different morphology when compared to the control HaCaT cells (Figure 6C). After 2.5 h of treatment, the number of viable cells continued to decrease (Figure 6F) and the very few viable cells were not found in their optimal conformation. Hence, both the MTT viability assay and the JuLI Stage observation demonstrated that the juniper treatment was toxic for the HaCaT cells in a time-dependent manner.

### 2.6. Evaluation of PNT1a Cells’ Viability after Exposure to Juniper EO MNDs

**Figure 7:**
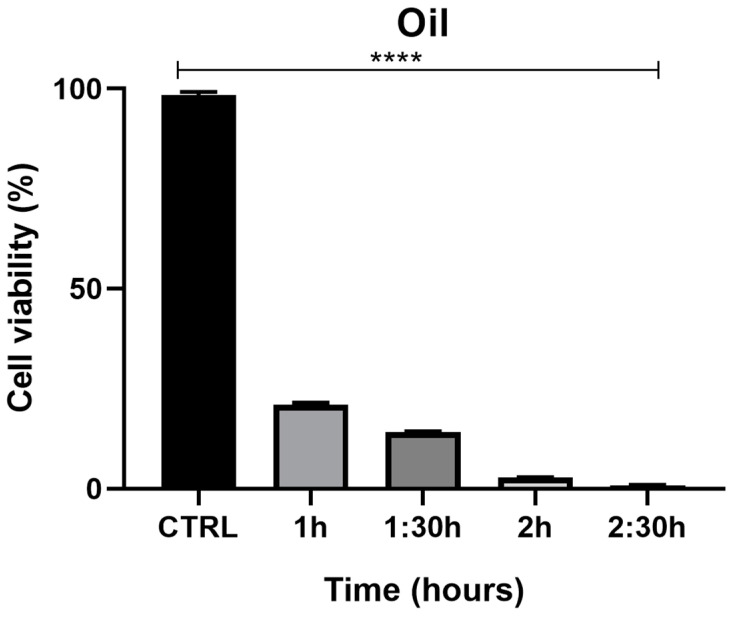
Effects of juniper MNDs on PNT1a cell viability. Figure illustrates a summary graph of the MTT assay conducted on PNT1a cells exposed to juniper EO MNDs at different times: 1 h, 1.5 h, 2 h, 2.5 h. Cell viability is represented as a percentage. The graph reveals significant differences in cell viability based on EO exposure time, with higher cell viability corresponding to shorter oil exposure times and maximum cell toxicity observed at the longest exposure time (2.5 h). Statistical analysis was performed by analysis of variance with Bonferroni corrections (**** *p* < 0.0001).

**Figure 8:**
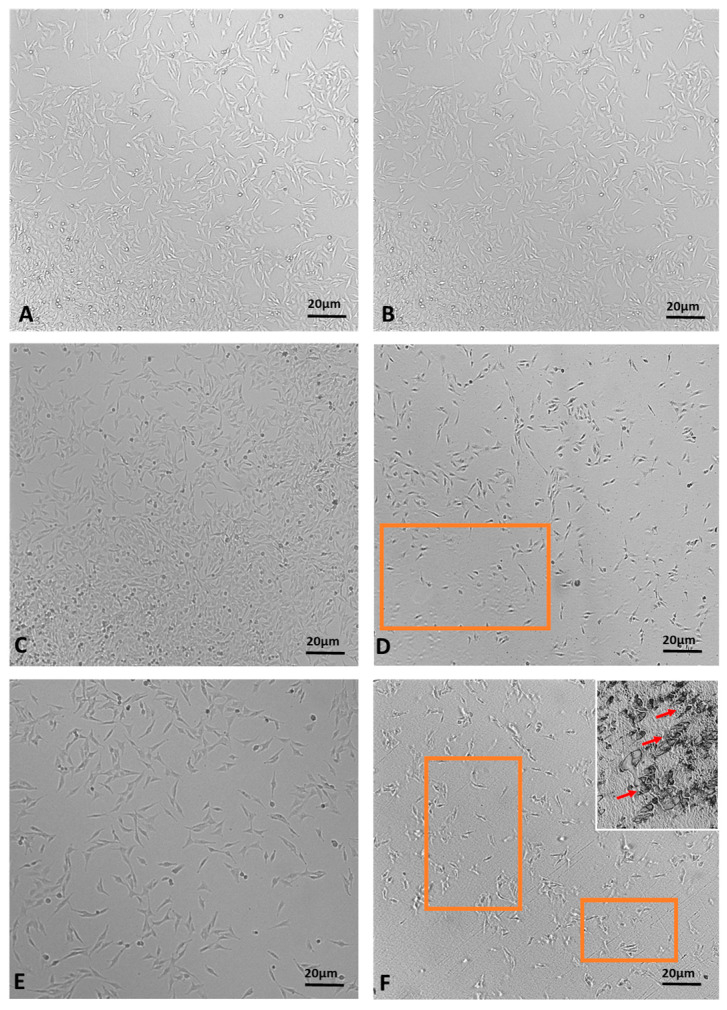
Time-lapse images of PNT1a cells during juniper EO MNDs treatment. (**A**,**B**) Control cells at time zero and after 2.5 h. (**C**,**D**) Cells at time zero, before adding juniper MNDs, and after 1 h of treatment. (**E**,**F**) Cells at time zero, before adding juniper EO MNDs, and after 2.5 h of treatment; in (**F**) (top right), the residue of the juniper EO microneedles/oil is visible. The orange boxes represent areas with decreased cell numbers. Arrows indicate juniper microneedles/oil. Scale bars: 20 µm.

The MTT assay demonstrated a time-dependent reduction in prostate cell (PNT1a) viability, decreasing from 22% at 1 h to 1.3% at 2.5 h (Figure 7). The viability percentage was calculated according to the following formula: (OD [570 nm] evaluated sample/(OD [570 nm] negative control) = R; R × 100 = % cell viability. Images of PNT1a cells captured with the JuLI Stage system confirm the data recorded from the MTT viability assay (Figure 8). In the control, after 2.5 h, cultured cells are similar to freshly plated cells (Figure 8A,B). One hour after adding the patch containing the MNDs with juniper EO to the plate, the number of cells clearly decreases compared to the freshly plated cells (Figure 8C,D). After 2.5 h from the beginning of the treatment, the viable cells are decimated (Figure 8E,F). In the insert at the top right of Figure 8F, it is possible to notice a deposit/accumulation of juniper MNDs/oil which mostly occupies the surface, taking the place of the cells. The images also demonstrate the alteration of cell morphology due to juniper EO MNDs; the cells, in fact, become thinner, losing the classic elongated shape typical of PNT1a cells.

## 3. Discussion

The main constituents of the extracted EO were α-pinene (>50%), ß-pinene (>10%) and limonene (>10%). In comparison with other Juniper genus EO the main constituents of *Juniperus communis* L. EO were dominated by α-pinene (51.4%), ß-pinene (5%), and limonene (5.1%) [24], while the main compositions of the *Juniperus communis* ssp. *hemisphaerica* were sabinene (25.1%) and α-pinene (13.6%) [25]. When our results were compared with the literature, several variations in the chemical composition of *Juniperus oxycedrus* ssp. EOs emerged. The berry oil from Spain, as described by Guerra Del Carmen and Garci in 1987, predominantly contained α-pinene at 60.6% and myrcene at 24.9% [26]. Moving to Croatia, Milos and Radonic’s study in 2000 reported the EO of *J. oxycedrus* berries to have α-pinene as the main compound at 66.3%, accompanied by an unidentified sesquiterpene hydrocarbon at 9.8%, β-myrcene at 4.9%, α-humulene at 1.1%, along with bornyl acetate and γ-cadinene each at 1.3% [27]. Angioni et al., in 2003, focused on the *J. oxycedrus* ssp. oxycedrus, finding the EO to be heavily dominated by α-pinene at 84.5%, followed by δ-3-carene at 3.6%, limonene at 2.5%, and myrcene at 2.1% [8]. In Lebanon, Loizzo and colleagues, in 2007, identified the *J. oxycedrus* ssp. oxycedrus berry EO as primarily composed of α-pinene at 27.4%, β-myrcene at 18.9%, α-phellandrene at 7.1%, and limonene at 6.7% [28]. Lastly, the work of Velasco-negueruela et al., in 2003, showed that the berry oils of *J. oxycedrus* ssp. badia were characterized by high amounts of α-pinene, ranging from 61.5% to 59.8%, and myrcene between 18.6% and 18.5%, along with smaller quantities of germacrene D and manoyl oxide [29]. These findings highlight the rich diversity in the chemical profile of *Juniperus oxycedrus* EO across different geographic regions and subspecies.

In this investigation, we utilized juniper EO, encapsulated within microneedles. This oil, extracted from the aerial parts of *Juniperus oxycedrus* L. ssp macrocarpa. Juniper EO is well known for its broad-spectrum antibacterial properties against strains like *S. aureus* and *P. aeruginosa*, as well as antifungal effects against *C. albicans* [22]. Furthermore, the application of juniper EO MNDs demonstrated equal efficacy in healing between bacterial and fungal infections, indicating a consistent and robust therapeutic outcome. In addition, the assessment demonstrated the absence of skin irritation after juniper EO MND application. Studies have also explored MNDs loaded with extracts from Chinese herbs, such as *Premna microphylla* and *Centella asiatica*, for treating wounds infected with *E. coli* and *S. aureus*. Notably, by the ninth day post-treatment, the wounds treated with these MNDs were significantly smaller compared to those in the control group [30]. Another experiment employed MNDs imbued with antibiotics like azithromycin and erythromycin, aiming to enhance deep tissue delivery. Histopathological examination revealed that wounds treated with azithromycin improved noticeably within five days, whereas erythromycin required a longer period [31]. For chronic wound infections, the antimicrobial medication must penetrate to reach and protect the viable cells while preventing the transport of exudates. MNDs excel in penetrating and dispensing active substances effectively at the site of application [11]. Importantly, our results on temporal killing activity also confirmed the antimicrobial activity of juniper EO and the successful release of this EO through the biodegradable formulation of the MND system.

To assess the wound healing efficacy of juniper EO MNDs, epithelial cells such as HaCaT and PNT1a were utilized. We have previously investigated the cytotoxic effects of juniper EO on embryonic fibroblast cells (NIH/3T3), observing a significant threshold (>32% *v*/*v*). Intriguingly, in vitro results indicate that juniper oil exhibits cytotoxicity towards both epithelial cells and microbes. While seemingly paradoxical, this dual effect might explain the hastened wound healing observed in treated animals compared to control subjects. Specifically, the juniper oil may promote the shedding of the wound’s most superficial cells, thereby facilitating the advancement of deeper layers. A similar effect has recently been demonstrated for segmented filamentous bacteria, whose presence in the intestine causes an increased turnover of epithelial cells [32], or by some disinfectants such as Chlorhexidine (CHX), which through an increase in ROS is cytotoxic to both microbes and epithelial cells, accelerating wound healing [33]. This cytotoxic action’s intensification over time might be attributed to the increased release of juniper constituents from the MND patch. Therefore, MNDs stand out as an ideal, minimally invasive strategy for precise drug delivery in wound management, effectively addressing the complexities of treating such conditions. In addition, the notable absence of adverse skin reactions affirms the favorable safety profile of juniper EO MNDs, supporting their potential use in therapeutic applications without inducing skin irritation. This safety profile underscores the biocompatibility of juniper EO MNDs and their suitability for therapeutic applications, complementing their wound healing efficacy. Finally, this finding is crucial for advancing the understanding of juniper EO MNDs as a safe and effective modality for dermatological applications.

## 4. Materials and Methods

### 4.1. Plant Material Treatment and Oil Extraction

In April 2021, Mr. Salvatore Mura, proprietor of “Fragus e Saboris de Sardigna” farm, harvested the above-ground parts of *Juniperus oxycedrus* L. ssp. macrocarpa. The collection took place in Sadali, a locality situated within south-eastern Sardinia’s Barbagia di Seùlo region, at coordinates 39_48049.2400 N, 9_16025.8000 E. A selection of these plant samples was archived in the Herbarium S.A.S.S.A. (recorded by M.U.; serial number: 16529) at the Department of Chemistry and Pharmacy at the University of Sassari. Aerial parts were gathered from Sadali (37′30° N, 11′03° E, altitude: 29 m) and were available in this period of the year. The samples were dried in airy premises, shielded from the light, then packed in paper bags and kept in the shade. Botanical voucher specimens were deposited in the herbarium of the Pharmacognosy laboratory of the Faculty of Pharmacy. For oil extraction, 10 kg of this plant batch was processed through a 3 h hydrodistillation using a custom-built extractor. The extraction yield varied from 0.17% to 0.18% by weight. Following extraction, the oils were decanted from the aqueous phase and stored at −20 °C for subsequent analysis. Adherence to the Italian Pharmacopeia 2008 standards ensured the essential oil (EO) composition and extraction efficiency. Using 300 g of plant material and a 4 h hydrodistillation in a Clevenger-type apparatus (Letslab^®^, Barcelona, Spain), the EO yielded 0.19–0.20% by weight. The EO was then dried following established methods using anhydrous sodium sulphate and stored at −20 °C for future analysis.

### 4.2. Oil Quantification and Analysis

For the quantification and analysis of essential oils, triplicate assays were performed on each specimen using a Hewlett Packard, Ramsey, MN, USA, Model 5890A gas chromatography (GC) setup. This device is equipped with a flame ionization detector and is coupled with a ZB-5 fused silica capillary column measuring 60 m in length and 0.25 mm in diameter with a 0.25 µm film thickness, supplied by Phenomenex. The specific GC procedural details are documented in a prior publication [34]. Quantification of the individual constituents was conducted by weight percentage, utilizing an internal standard—2,6-dimethylphenol—and incorporating response factors to ensure accuracy. Further, GC coupled with mass spectrometry (GC/MS) analysis was executed using an Agilent Technologies model 7820A integrated with a 5977E MSD mass selective detector from the same manufacturer, adhering to the above-mentioned conditions and column specification. Mass scanning was performed within the range of 10–900 AMU at an ionization energy of 70 eV. For the identification phase, mass spectral peaks within the range of 40–900 AMU were analyzed. The identification of compounds was based on matching their retention times with those of known standards and interpreting their electron ionization (EI) fragmentation patterns. Compounds were identified by matching their mass spectra and retention times with those reported in the literature: the NIST98 (NIST/EPA/NIH, 1998), FLAVOUR, and LIBR (TP) (Adams, 2004) mass spectra libraries were used as references.

### 4.3. Microneedle Preparation

Poly(vinyl alcohol) (PVA), with a molecular weight (Mw) of 31,000–50,000, and 87–89% hydrolyzed polyvinylpyrrolidone (PVP), Mw = 58,000, (Plasdone^TM^ K-29/32, Ashland, Paterson, NJ, USA), were purchased from Sigma-Aldrich, St. Louis, MO, USA and Ashland, Wilmington, DE, USA, respectively. Tween^®^ 80 was obtained from VWR Chemical, LLC, Solon, OH, USA. Juniper essential oil was used to fabricate oil-based MNDs. Firstly, a mixture of PVA (15% *w*/*w*) and PVP (20% *w*/*w*) (PP2) was prepared by mixing pre-prepared PVA (30% *w*/*w*) and PVP (40% *w*/*w*) gels at a ratio of 1:1. Afterwards, 8 g of PP2, 1.6 g of juniper EO, and 160 mg of Tween 80 were weighed and vigorously mixed using a speed mixer at 3000 rpm for 5 min in a homogeneous gel mixture. Then, 500 mg of the resulting gel mixture was added to each silicon mold and centrifuged at 5000 rpm for 15 min. Finally, the gel-filled molds were then allowed to solidify in an ambient environment for 48 h. The sidewalls of the final dried MAPs were removed using scissors before being stored in a desiccator for further use.

### 4.4. Yeast and Bacterial Strains’ Isolation and Identification

The *C. albicans* strain was isolated from clinical specimens of blood infection at Mowasat Hospital in Damascus, then cultured on Sabouroud dextrose agar medium (Thomas Scientific, Swedesboro, NJ, USA) for 24 h at 37 °C. Confirmation of *C. albicans* was achieved through a Gram test and subsequent cultivation on blood agar. Following cultivation, cells were harvested, and the DNA was extracted [35]. Multiplex PCR was performed using yeast specific universal primer UNI1 and UNI2. In addition, the species-specific primer Calb was used based on the sequence data for the ITS1 and ITS2 regions of the reference strains and clinical isolates from the candida genus available in the EMBL/Genebank database. The specific primers used are described in the Supplementary Materials (Table S2). *P. aeruginosa* and *S. aureus* were isolated from clinical samples of blood infection at Mowasat Hospital in Damascus. These strains were cultured on nutrient broth for 7 days at 24 h, blood agar (King medium B base, HIMEDIA, Modautal, Germany) and manitol salt agar (HIMEDIA, Modautal, Germany) and subjected to a Gram test (Thomas Scientific, Swedesboro, NJ, USA). gGenomic DNA (gDNA) was extracted using MolYsis™Basic (Molzym GmbH & Co., KG, Bremen, Germany). PCR reactions on the gDNA were performed using three pairs of primers as described in the Supplementary Materials (Table S3). The first pair of primers were used to amplify 16SDNA to confirm the bacterial infection. The second pair of primers targeted a 189 bp region specific for the Pseudomonas genus (SSS), and the third pair amplified a 359 bp region specific for Staphylococcus genus (aur). All PCR reactions were carried out using a thermal cycler (Clever, Rugby, UK).

### 4.5. Evaluation of Time-Dependent Antimicrobial Activity

The time-dependent antimicrobial efficacy of juniper EO MNDs was assessed by incubating the MNDs with bacterial strains (*P. aeruginosa* and *S. aureus* at a concentration of 2 × 10^8^ CFU/mL and *C. albicans* at 2 × 10^7^ CFU/mL) in LB broth at 37 °C. The concentration of EO used was 32 *v*/*v*%, determined based on the MICs obtained from our previous study [36]. Finally, the CFUs were quantified at various time intervals (4 h, 24 h, 48 h) during the incubation period.

### 4.6. Evaluation of Wound Healing with MND-Assisted Application of Juniper EO in Infected Rats

Male rats weighing 200 g were provided by the Faculty of Biology at Damascus University. The laboratory strictly adhered to laboratory animal care guidelines, and all animal procedures were approved by the Ethics Committee of Damascus university with ID number (ID: SNV-241023-136), following the principles outlined in the UK. Animals (Scientific Procedures) Act, 1986, and associated guidelines from EU directive 2010/63/EU for animal experiments. All rats were initially anesthetized with chloroform (Merck) by replacing animals in a closed container. The wounds, 1 cm in diameter, were created on the backs of rats using sterilized surgical scissors. A volume of 100 µL of the infecting solution was prepared at concentrations of 2 × 10^8^ CFU/mL for *P. aeruginosa* and *S. aureus* and 2 × 10^7^ CFU/mL for *C. albicans*. This infecting solution was applied to the wound area using a sterilized syringe to induce severe bacterial and fungal infections. The rats were divided into three groups, each infected with different microorganisms to mimic bacterial and fungal infections. One microorganism (*P. aeruginosa* or *S. aureus* or *C. albicans*) was used for each group. Subsequently, MNDs loaded with juniper EO were applied to the wound areas at 4 h post-infection and throughout the course of treatment. A group of uninfected wounds served as a negative control for infection and a group of untreated infected wounds acted as the negative control (baseline) for MNDs loaded with juniper EO application, reflecting the real-world comparisons of novel treatments to existing standards or no treatment. Gentamicin-treated wounds were employed as a positive control for bacterial infection, and fluconazole-treated wounds served as a positive control for fungal infection. The healing process of each group was systematically monitored and assessed at different time points (3, 6, 9, and 12 days) following MND application.

### 4.7. Histological Assessment of Wound Healing

Skin tissue excised at wound areas was fixed in 10% formalin, followed by tissue processing, paraffin blocking, and cutting into 5 µm thin sections using a microtome. Staining was conducted using the hematoxylin and eosin (H&E) stainingmethod, and the prepared slides were examined under a light microscope. On days 3, 9, and 12, images of the wounds were captured using a digital camera attached to the microscope. The tissue response to various treatments at different time intervals was recorded.

### 4.8. Cell Culture

Two distinct cell lines were employed for this study. HaCaT cells, immortalized keratinocytes, were obtained from the A.T.C.C. (American Type Culture Collection, Manassas, VA, USA), while PNT1a cells (human prostate cell line derived from the immortalization of adult prostate epithelial cells) were obtained from the E.C.A.C.C. (European Collection of Cell Culture, Salisbury, UK). The HaCaT cells were cultured in Dulbecco’s Modified Eagle’s Medium (DMEM, Sigma-Aldrich) supplemented with 10% fetal bovine serum (FBS, Sigma-Aldrich) and a mixture of penicillin and streptomycin (Sigma-Aldrich). This culture was maintained at 37 °C in a humidified incubator with 5% CO_2_. PNT1a cells were cultured individually in Roswell Park Memorial Institute (RPMI, Sigma Aldrich) medium with the addition of 10% fetal bovine serum (FBS, Sigma), 1% L-glutamine (Sigma), and 2% penicillin/streptomycin (Sigma) in a 37 °C incubator with 5% CO_2_ and controlled humidity. The cells were detached enzymatically with 0.25% trypsin/EDTA when the cells reached 70% confluence. All cells were analyzed morphologically using a cell imaging system (JuLI Stage microscope, Nano Enteck, Waltham, MA, USA).

### 4.9. MTT Assay

The MTT assay was performed to assess the effects of juniper EO MNDs on the two cell lines at different time intervals. Cells (30,000 per well) were gently dislodged with phosphate-buffered saline (PBS, Sigma; D8537-500ML) and trypsin then placed in 24-well plates in 500 µL of complete culture medium (DMEM for HaCaT and RPMI for PNT1a) for 24 h. After adhesion, the culture medium was gently removed and replaced with white DMEM medium and white RPMI with 1% FBS, for HaCaT and PNT1a, respectively, as previously described [37]. The cells were then treated with patches caring juniper MNDs, achieving an oil concentration of 32 *v*/*v*% according to MICs obtained from our previous work [38]. Samples were collected at various time intervals (1 h, 1.5 h, 2 h, 2.5 h). At the end of the treatment, 50 µL of MTT salt (Merck, M5655-500M MG) was added to each well (5 mg/mL) for 3.5 h. Following this, the culture medium was replaced with 500 µL of DMSO. The amount of formazan, presumed to be directly proportional to the number of viable cells, was measured by recording absorbance changes at 570 nm using a plate reader (Synergy HTX Multi mode microplate reader). The viability percentage was calculated according to the following formula: (OD [570 nm] evaluated sample/(OD [570 nm] negative control) = R; R × 100 = % cell viability. If the percentage was above 50%, the tested substance was considered non-cytotoxic, whereas values below 50% indicated cytotoxicity [38]. The Prestoblue test was performed in triplicate for each experimental class.

### 4.10. Statistical Analysis

All experiments were replicated a minimum of three times. Data analysis was conducted using Microsoft Excel (2010) and the Graphpad Prism software version 6 (GraphPad prism 6 software, Graphpad Software Inc., La Jolla, CA, USA). The results are presented as the mean plus standard deviation (SD) and analyzed by one-way or two-way ANOVA comparison tests followed by the appropriate correction, as specified in the caption under each figure. Values were considered statistically significant when *p* < 0.05.

### 4.11. Summary of Methodological Procedures

**Figure 9:**
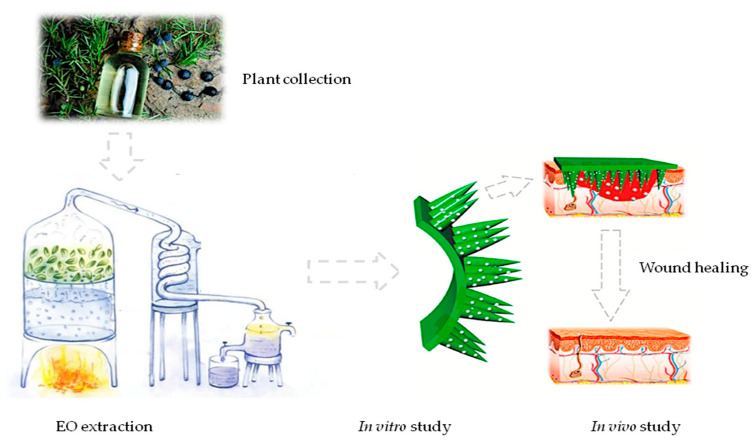
Methodological procedure summary. The figure shows a graphiclal summary of all methodological procedures.

The methodological procedure strated with plant collection and EO extraction followed by in vitro and in vivo studies (Figure 9).

## 5. Conclusions

The results of our study demonstrated that juniper EO MNDs were able to inhibit bacterial and fungal proliferation in vitro and in vivo models, thus preventing microbial infection at the site of the wound infection. Furthermore, it was also demonstrated that treatment of the infected wound with juniper EO MNDs was able to improve wound healing with quick tissue recovery. Finally, juniper EO MND transdermal patches could be a novel and safe strategy in the treatment of wounds infected with bacterial and fungal pathogens, and is considered as a promising approach to improve drug delivery across skin and prevent infection. More studies are needed, particularly in human, to confirm the efficacy of this technique in infected-wound healing.

## Data Availability

Data is contained within the article and Supplementary Material.

## Supplementary Materials

The following supporting information can be downloaded at: https://www.mdpi.com/article/10.3390/ph17010040/s1, Table S1: Chemical composition of Sardinian *Juniper oxycedrus* L. ssp. Macrocarpa essential oil; Table S2: Universal and species-specific primers used in *C. albicans* molecular identification; Table S3: Primers used in PCR for bacterial molecular identification.
