# Supplementary material for: Juniperus oxycedrus L. ssp. Essential Oil Microneedles: A Promising Antimicrobial and Wound Healing Activity
Source: Pharmaceuticals (Basel). 2023 Dec 27;17(1):40. doi: 10.3390/ph17010040 (PMC10821373; doi:10.3390/ph17010040)
Supplement: Supplementary file 1 [file pharmaceuticals-17-00040-s001.zip › pharmaceuticals-2755767-supplementary.pdf]

Supplementary Materials

Table S1. Chemical composition of Sardinian *Juniper oxycedrus* L. ssp. Macrocarpa essential oil. The table includes the chemical composition of essential oil from aerial parts of Sardinian *Juniper oxycedrus* L. ssp. Macrocarpa based on GC/MS analysis. Column was a "no-polar column ZB-5". Data represent the mean of three replicates ± SD.

Rt	RI apol Lett	RI apol Sper	Constituents	%	I.D.[(a)]
10.38	700	704	heptane	0.05±0.01	RI, MS
21.50	920	920	ß-thujene	0.10±0.02	Std
22.08	937	939	α-pinene	56.63±0.24	Std
22.89	945	953	α-fenchene	0.05±0.01	Std
23.01	956	955	camphene	1.50±0.02	Std
23.59	970	974	benzaldehyde	0.04±0.01	RI, MS
24.14	975	977	α-sabinene	0.33±0.03	Std
24.59	979	981	ß-pinene	13.42±0.09	Std
24.90	991	992	ß-myrcene	0.73±0.04	Std
25.94	1004	1003	pseudolimonene	0.03±0.01	RI, MS
26.06	1003	1005	α-phellandrene	0.04±0.01	Std
26.22	1008	1011	γ-3-carene	0.66±0.02	Std
26.64	1017	1015	α-terpinene	0.30±0.02	Std
27.06	1025	1026	p-cymene	0.51±0.03	Std
27.36	1029	1027	limonene	14.66±0.11	Std
27.46	1026	1030	benzyl alcohol	3.41±0.05	RI, MS
27.59	1026	1031	1,8-cineole	1.37±0.03	Std
28.88	1060	1064	γ-terpinene	0.16±0.02	Std
30.39	1088	1087	γ-terpinolene	0.14±0.01	Std
32.46	1129	1128	*cis*-allo-ocimene	3.00±0.07	RI, MS
33.16	1131	1131	*trans*-allo-ocimene	0.25±0.03	RI, MS
33.59	1137	1141	*trans*-sabinol	0.06±0.01	RI, MS
35.17	1169	1166	endo-borneol	0.03±0.01	RI, MS

35.50	1177	1180	terpinen-4-ol	0.13±0.02	Std
35.67	1179	1183	p-cymen-8-ol	0.02±0.01	RI, MS
36.13	1186	1180	α-terpineol	0.08±0.02	Std
39.99	1189	1287	bornyl acetate	0.08±0.01	RI, MS
42.39	1350	1352	α-cubebene	0.03±0.01	Std
42.73	1350	1353	α-longipinene	0.02±0.01	RI, MS
45.06	1419	1419	ß-caryophyllene	0.40±0.08	Std
46.17	1452	1454	humulene	0.07±0.02	Std
46.58	1480	1480	γ-muurolene	0.03±0.01	RI, MS
46.89	1485	1482	germacrene D	0.05±0.01	Std
47.78	1521	1523	δ-cadinene	0.10±0.02	Std
47.93	1529	1530	calamenene	0.02±0.01	RI, MS
49.81	1582	1583	caryophyllene oxide	0.07±0.02	Std
			Total	99.72	
			Hydrocarbon monoterpene	72.5	
			Oxygenated monoterpene	1.28	
			Hydrocarbon sesquiterpenes	23	
			Oxygenated sesquiterpenes	2	
			Diterpenes	0.54	
			Others	0.4	

(a) Identification methods: RI (Retention Index): Comparative analysis with retention indexes reported in the literature; Std (Standard): Evaluation based on comparison of the retention time and mass spectrum with available authentic standards; MS (Mass Spectrum): Comparison with computer mass libraries such as Adams, Nist 11 and by interpretation of the mass spectra fragmentations.

Table S2. Universal and species specific primers used in *C. albicans* molecular identification.

**Species**	**Primer name**	**Sequences**
Clinically relevant Yeasts	UNI 1<br>UNI 2	GTCAAACTTGGTCATTTA<br>TTCTTTTCCTCCGCTTATTG
C. albicans	Calb	AGCTGCCGCCAGAGGTCTAA

Table S3. Primers used in PCR for bacterial molecular identification. The table shows all primers that used in the PCR reaction for *P. aeruginosa* and *S. aureus* molecular identification and the amplified gene for each genus and the length of the amplified fragment in bp

**Species**	**Primer name**	**Sequences**
16S rDNA	*16S*	AGAGTTTGATCMTGGCTCAG<br>GYTACCTTGTTACGACTT
P. aeruginosa	*SSS*	GCCTCTACCAGTACCTGCTAC<br>GYTACCTTGTTACGACTT
S . aureus	*aur*	TCGCTTGCTATGATTGTGG<br>GCCAATGTTCTACCATAGC
